# A three-group study, internet-based, face-to-face based and standard- management after acute whiplash associated disorders (WAD) – choosing the most efficient and cost-effective treatment: study protocol of a randomized controlled trial

**DOI:** 10.1186/1471-2474-10-90

**Published:** 2009-07-22

**Authors:** Anne Söderlund, Annika Bring, Pernilla Åsenlöf

**Affiliations:** 1 Department of Physiotherapy, School of Health, Care and Social Welfare, Mälardalen University, Västerås, Box 833 SE-721 23 Västerås, Sweden; 2 Section of Physiotherapy, Department of Neuroscience, Uppsala University, Akademiska Hospital, ING 15, SE-751 85 Uppsala, Sweden

## Abstract

**Background:**

The management of Whiplash Associated Disorders is one of the most complicated challenges with high expenses for the health care system and society. There are still no general guidelines or scientific documentation to unequivocally support any single treatment for acute care following whiplash injury.

The main purpose of this study is to try a new behavioural medicine intervention strategy at acute phase aimed to reduce the number of patients who have persistent problems after the whiplash injury. The goal is also to identify which of three different interventions that is most cost-effective for patients with Whiplash Associated Disorders. In this study we are controlling for two factors. First, the effect of behavioural medicine approach is compared with standard care. Second, the manner in which the behavioural medicine treatment is administered, Internet or face-to-face, is evaluated in it's effectiveness and cost-effectiveness.

**Methods/Design:**

The study is a randomized, prospective, experimental three-group study with analyses of cost-effectiveness up to two-years follow-up. *Internet – based programme *and *face-to-face group treatment programme *are compared to *standard-treatment *only. Patient follow-ups take place three, six, twelve and 24 months, that is, short-term as well as long-term effects are evaluated. Patients will be enrolled via the emergency ward during the first week after the accident.

**Discussion:**

This new self-help management will concentrate to those psychosocial factors that are shown to be predictive in long-term problems in Whiplash Associated Disorders, i.e. the importance of self-efficacy, fear of movement, and the significance of catastrophizing as a coping strategy for restoring and sustaining activities of daily life. Within the framework of this project, we will develop, broaden and evaluate current physical therapy treatment methods for acute Whiplash Associated Disorders. The project will contribute to the creation of a cost-effective behavioural medicine approach to management of acute Whiplash Associated Disorders. The results of this study will answer an important question; on what extent and how should these patients be treated at acute stage and how much does the best management cost.

**Trial registration number:**

Current Controlled Trials ISRCTN61531337

## Background

Whiplash is an acceleration-deceleration mechanism of energy transfer to the neck, which may result in bony or soft-tissue injuries mostly in motor vehicle accidents. This trauma can cause a multitude of clinical symptoms, known as Whiplash Associated Disorders (WAD) [1]. While other personal injuries are decreasing in incidence, neck injuries continue to increase. Number of persons seeking care for traffic related neck injuries has increased in annual cumulative incidence from 83 per 100,000 inhabitants (1985–1986) to 302 per 100,000 (1997–1998) [2]. Also, health care costs for pain patients are increasing. The total cost for Swedish society for sick leave, physician visits, rehabilitation, early retirement pension, etc., in 2003 amounted to SEK 87,5 billion crowns for people with chronic pain [3].

The management of WAD is one of the most complicated challenges for the health care system and society. According to tradition, acute pain is considered as a consequence or a symptom of a patho-physiological lesion, and is analysed and treated accordingly. However, psychosocial factors often complicate the situation at early stage building a platform where acute pain might transform to chronic. There are still no general guidelines or scientific documentation to unequivocally support any single treatment for acute care following whiplash injury. Barnsley et al. [4] concluded, however, that early intervention intended to reduce the number of chronic cases could be particularly worthwhile. A Swedish study [5] showed that early active intervention leads to fewer people on sick leave and fewer chronic cases in patients with acute musculoskeletal pain.

Clinical experience in Sweden has shown that about one third of acute whiplash patients develop chronic problems. This is consistent with international studies in which 20 to 40 percent of all patients with whiplash injuries develop chronic problems [6]. Clinical practice in the acute phase includes several strategies, of which exercise therapy is the most common, though a review of the literature shows few controlled studies of this form of treatment [7-12]. The treatments in these studies of acute WAD included only management of physical and medical symptoms. However, neck pain can affect functional capacity and be related with psychological factors such as catastrophizing, fear of movement, and self-efficacy. Specific effects of these psychological factors during the course of illness in WAD have only been partially studied [11,13-18].

Catastrophizing refers to an exaggerated negative interpretation of pain and health problems in general [19]. In several studies catastrophizing has been associated with increased depression, emotional distress, physical dysfunction, health care use and pain experience [17,20]. In a study of coping process over time in patients with WAD it was shown that catastrophic thinking appeared at six weeks follow up after the injury [17]. Catastrophizing can also be related to fear-avoidance behaviour [21]. Avoidance leads to sustained and worsened fear, which in turn leads to a reduction of physical and social activity [22]. A more specific fear is a fear of movement where the patient incorrectly believes that physical activity would worsen health. Such specific fear can also be an important predictor for long-term health problems and limitation of activity [23].

A concept deemed to be one of the most important in self-management of health problems is self-efficacy. Self-efficacy expectancies are defined as a personal belief of how successfully one can cope with difficult situations [24]. Individuals with high self-efficacy expectations should thus be more persistent in difficult situations [24] and perceive their disabilities less severe than the ones experiencing low self-efficacy [11,25]. There is increasing evidence that self-efficacy plays an important role in rehabilitation processes [26,27]. Therefore, self-efficacy should be an explicit goal of rehabilitation aimed to increase person's self-management skills [27,28] and in increasing individual's physical activity level [29].

The predictive validity of psychosocial factors in disability such as those above have been studied quite extensively. However, studies with experimental design needed to elucidate if it is possible to influence these factors in a treatment context are rare. An alternate perspective on pain with a keystone on behavioural learning that acknowledges among other things the abovementioned psychological and social factors is worth attention in WAD research. An integration of the medical and behavioural perspectives i.e. behavioural medicine offers an understanding of the individual experiences and consequences of pain. It also provides a basis for the development of interventions targeting prevention of chronic disabling pain as well as self-management of pain. However, research support for this approach is yet inadequate. The behavioural medicine treatment has previously been studied in patients with musculoskeletal pain and was found to be more effective than physical exercise alone [27]. This new self-help management will concentrate to those psychosocial factors that are shown to be predictive in long-term problems in WAD, i.e. the importance of self-efficacy, fear of movement, and the significance of catastrophizing for restoring and sustaining activities of daily life.

Behavioural medicine treatment over the Internet (IT) is a fairly new form of self-help management. Its successful effectiveness has been investigated in studies on chronic back pain [30], recurrent headache [31], tinnitus [32], panic disorder [33] etc. The results from these studies indicate that the Internet is a cost-effective management and as effective as face-to face treatment. The mean age for the patients with acute WAD is mostly about 40 years [1,2] they are working and busy with their daily life. Thus, it is reasonable to think that a treatment that could be delivered at any chosen time of a day would be appropriate. Therefore, an Internet based treatment is worthwhile to try for a larger group of patients with WAD. To our knowledge IT- based behavioural medicine self-help treatment has not been developed and tested on WAD.

### Hypothesis

The main purpose of this study is to try a new behavioural medicine intervention strategy at acute phase aimed to reduce the number of patients who have persistent problems after the whiplash injury. The goal is also to identify which of three different interventions that is most cost-effective for patients with Whiplash Associated Disorders. In this study we are controlling for two factors. First, the effect of behavioural medicine approach is compared with standard care. Second, the manner in which the behavioural medicine treatment is administered, Internet or face-to-face, is evaluated in it's effectiveness and cost-effectiveness.

## Methods/design

The following CONSORT recommendations for reporting RCT [34,35] are used to enable readers understanding of the present study protocol and the course of the study.

### Study Design

**Figure 1:**
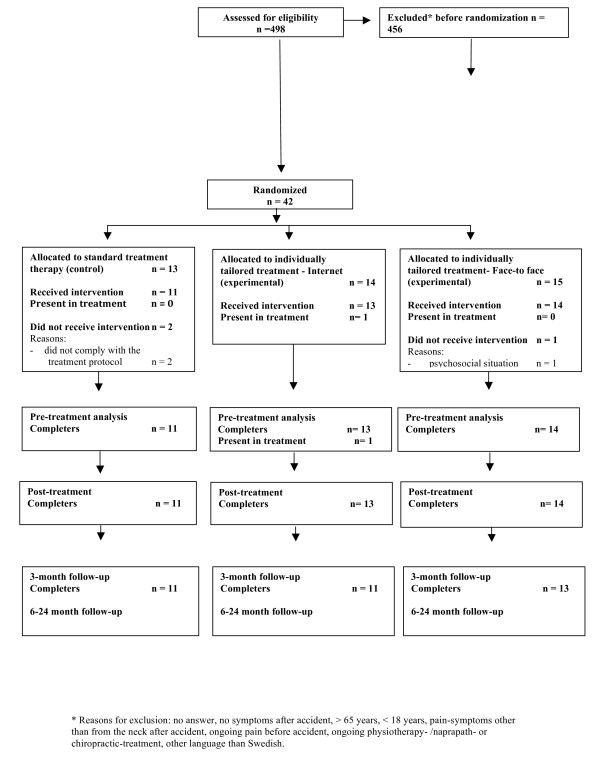
**CONSORT-diagram over the participant flow in the study**. CONSORT-diagram over the participant flow through recruitment, randomization, baseline measurement, immediately post-treatment, and 3–24-month follow-up in the WAD-IT study.

The study is a randomized, prospective, experimental three-group study with analyses of cost-effectiveness up to two-years follow-up (see Figure 1). IT- based programme (Group 1) and face-to-face group treatment programme (Group 2) are compared to standard-treatment only (Group 3) Patient follow-ups take place three, six, twelve and 24 months, that is, short-term as well as long-term effects are evaluated. Figure 1 presents the study design and patients' flow till now.

### Study setting and Participants

Patients will be enrolled via the emergency ward during the first week after the accident. All patients get a letter from the emergency staff during they visit that explains the reason why they will be contacted by a physiotherapist. The emergency staff will also give standard treatment instructions for all patients. The second author will approach the patients by phone after receiving a list of patients that seek the emergency ward. During the phone call an informed consent is received. All eligible patients are scheduled to an appointment with the calling physiotherapist. During the appointment the patients that fulfil inclusion criteria will complete the baseline measures. Inclusion criteria for this study are age between 18 and 65, and satisfactory Swedish language skills. Patients are to fulfil criteria for the diagnosis of WAD grade I and II [1] and have ongoing pain in the neck due to the accident. Participants should also have access to a computer to be eligible for the study. Patients with prior neck injury, other ongoing chronic pain problems or ongoing treatment for pain or pain related symptoms are not eligible.

### Randomization

After completion of baseline measures, patients are randomly allocated to one of the three groups using blocked cluster randomization. Patients are randomized in random blocks of 9 and 18 so that three consequent patients will always be distributed into the same treatment group. This strategy is used to obtain homogeneity in face-to-face treatment groups regarding number of days since the accident. The randomization list has been conducted in advance with a computer programme by a statistician and the therapist is blind for the upcoming blocks.

### Intervention

All three groups receive "standard care as usual" before randomization, i.e. patients get a home exercise programme at the emergency ward. All treatments are provided by one therapist (the second author).

The experimental intervention is based on an incorporation of perspectives from medical and behavioural sciences. The fundamental principle of the program is a theoretical understanding of human behaviour and individual's motivation for health behaviour change [36]. The main clinical philosophy behind the program is to guide patients toward resumed activities of everyday life. Functional behavioural analyses and personal treatment goals are established to identify the physical, cognitive, and behavioural skills necessary for goal achievement [27].

Group 1 and 2 treatments are individually tailored behavioural medicine intervention targeting physical, behavioural, and psychosocial factors of relevance for pain adjustment. A recently developed treatment protocol [27] has been modified and is applied in the study. This behavioural medicine approach includes seven phases and was organized in a cognitive-behavioural framework; 1. Behavioural goal identification [37], i.e. identification of activities that are problematic and highly prioritised for the patient, 2. Self-monitoring of these activities, 3. Individual functional behavioural analysis, 4. Basic skill acquisition, 5. Applied skill acquisition, 6. Generalisation, and 7. Maintenance and relapse prevention. These phases are divided in seven treatment modules (A – G) (see Additional file 1).

Tailored treatment strategies related to each chosen goal activity include skills training to increase self-efficacy, decrease the impact of psychological risk factors, e.g. catastrophic thinking and fear of movement. Also, physical skills acquired in activity performance are taught. Patients are educated in problem solving skills for daily activities. Self-management of symptoms through using these new psychosocial tools is emphasised as are problem-solving skills related to new activities and every day life situations. In the final phase a written summary of the treatment is elaborated together with personal relapse prevention strategies.

#### Group 1

The new IT-based treatment (Internet/e-mail) regimen for acute WAD patients, emphases self-monitoring and skills training, as well as discussions led by a therapist. The patient will be instructed when to start and how to log onto the Internet treatment modules (seven modules; A – G), one at a time and get individualized support via e-mail from a therapist. The patient cannot log onto the next module if she/he has not completed the tasks in the previous module. Questions from the patients as well as answers from the therapist will be available on the treatment site, by consent from the patient, functioning as an additional support for the fellow patients, i.e. virtual group treatment.

#### Group 2

Face-to-face intervention involves groups of three to six patients led by therapists. The therapist meets with the group seven times during the acute phase following the accident. The face-to-face programme is planned to be similar to the IT-based treatment regimen described above, but differs in the way the treatment is administered. The programme consists of those seven treatment phases in seven modules described above (Additional file 1).

#### Group 3

Standard care of these patients currently involves a visit to a physical therapist, which provides a home exercise programme dealing with physical symptoms and advice of returning to normal activities as soon as possible. No further treatment is given except the home exercise programme that all patients get at the emergency ward (standard care) before randomisation.

### Treatment fidelity

Measurements of the independent variables, ie, strategies for enhancing treatment fidelity [38], are conducted during the course of the intervention. The following treatment fidelity strategies [38] are used:

1. To ensure the same treatment within condition a detailed treatment manual and a treatment protocol/checklist is used for each patient separately. The treatment manual has also been developed to guarantee that the treatment will be unchanged during the course of the study.

2. Both IT-based and Face-to-face-based interventions are going to have equal number of treatment sessions/phases to ensure equivalent dose across conditions.

3. Data expert support is available in case of implementation setbacks in the IT-group.

4. One therapist will deliver both group treatments to ensure standardized trained therapist and to minimize contamination between conditions.

5. Patients can e-mail their questions in IT-group to the therapist. These questions, we believe, will mirror patient's understanding of the treatment. Also, often asked questions and answers will be on the IT-group's home page for all patients in this group to read.

6. By asking so called consumer questions, we are recording patients' beliefs and expectations about the intervention and also, if the expectations are fulfilled.

7. To ensure that the patients are able to use cognitive and behavioural skills we are applying home exercises in daily activities. These are reported in a diary and always discussed with the patient.

8. To ensure that the behavioural components are not given to the standard care-group in the acute stage the therapist follows a strict manual only dealing with physical symptoms and advice given for all patients at the initial visit.

9. The number of intervention contacts (e-mail contacts and Face-to-face group meetings) is reported.

### Primary outcome

Primary outcome measure is disability measured with Pain Disability Index [39-41]. PDI is a 7-item inventory that is designed to measure interference with role-functioning due to persistent pain in the following areas: 1) family and home responsibilities, 2) recreation, 3) social activity, 4) occupation and education, 5) sexual behaviour, 6) self-care, and 7) life-support activity. Degree of interference is rated on 11-point numeric rating scales (NRS), ranging from 0 (no interference) to 10 (total interference). A total disability score range from 0 to 70. The PDI is found to be a reliable and valid measurement of disability in patients with persistent pain [39,41] as well as patients with acute pain [11]. A Swedish version of the PDI [25] is used in this project.

#### Cost accounting measures

Cost-effectiveness will be evaluated through direct and indirect costs during the first two years after a whiplash trauma. For direct medical costs analyses data from medical procedures, medication costs, general practice care and paramedical care are gathered from different sources; patients' medical records and self-reported data. Indirect costs (self-reported data) include loss of paid labour, i.e. total number of sick days and the proportion of patients receiving disability pension as well as compensations from insurance companies in form of invalidity percents. A cost-diary is used to measure direct and indirect health costs [42]. The patients fulfil a four-week diary. For each day the patients report costs form medical procedures, medication costs, general practice care and paramedical care. They also report any kind of costs for training that are due to their pain problems. All kind of help the patients have at home due to pain problems and total number of sick days is reported.

The cost diary has been translated and adjusted to the Swedish healthcare system and to possible administration via Internet.

### Secondary outcome

The following secondary outcome measures will be used in this study:

The Patient Goal Priority Questionnaire (PGPQ) is a patient-specific measure designed for behavioural goal assessment (i.e. to collect data concerning patients' priorities of behavioural goals) and for use as a tailored outcome measure. The PGPQ consists of two sections. In the first part of the questionnaire, patients report 1 to 3 activities that they: (1) are unable or have difficulties to perform due to pain, and (2) wish to influence with treatment. The relative importance of the activities is ranked by the patients, with 1 representing the most important activity. In the second part of the PGPQ, patients score current level of (a) behavioural performance on a numerical rating scale (NRS) ranging from 0 to 10 (high scores = severe limitations), (b) frequency of behavioural performance during the past week on an ordinal scale with five grades (0 = never, 1 = once, 2 = twice, 3 = three to five times, 4 = more than five times), (c) satisfaction with current level of behavioural performance on a NRS ranging from 0 to 10 (high score = high satisfaction), (d) self-efficacy for behavioural performance on a NRS ranging from 0–10 (high score = high confidence), (e) fear of behavioural performance on a NRS ranging from 0–10 (high score = high fear), (f) expectations of future behavioural performance as a result of treatment on a NRS ranging from 0–10 (high score = severe limitations), and (g) readiness to adopt new behaviours to attain expectations of behavioural performance on a NRS ranging from 0–10 (high score = high readiness). The above items are scored separately with regard to each of the ranked goal activities [37,43].

Tampa Scale for Kinesiophopia (TSK) [23], that measures fear of movement and (re)injury. The TSK consists of 17 items with a 4-grade format in which 1 strongly disagree and 4 strongly agree. A total score range from 17 to 68, in which a higher score indicates more fear. The Swedish version of TSK has shown good reliability in evaluating fear of movement and (re)injury in patients with musculoskeletal pain [25] and WAD [44].

Pain Intensity Diary [11], is used to measure pain intensity three times a day, four days. The Numerical Rating Scale is used where 0 implies no pain and 10 maximum pain.

Self-Efficacy Scale [11,13,25,28] (SES) is used to measure the strength of perceived self-efficacy in performing 20 common everyday life activities. Participants are asked to rate how confident they are to perform each of the 20 activities in spite of pain. The response format is 11-grade NRS in which 0 imply not at all confident and 10 very confident. A total self-efficacy score range from 0 to 200 was. The Swedish version of SES has shown good reliability in evaluating self-efficacy in patients with musculoskeletal pain [25] and WAD [11]

Coping Strategies Questionnaire [45,46], (CSQ) is a 48-item checklist where patients are asked to indicate the extent to which they use certain cognitive ('Reinterpreting pain sensations' (RPS), 'Coping self-statements' (CSS), 'Ignoring sensations' (IS), 'Diverting attention' (DA), 'Praying/hoping' (PH) and 'Catastrophizing' (Ca)) or behavioural ('Increased behavioural activity' (IBA) and 'Pain behaviours' (PB)) coping strategies. Items are summarised into eight subscales. The scores range from 0 to 6 for each item and the maximal score in each sub-scale is 36. Higher scores indicate that a person uses the particular coping strategy more extensively. The CSQ includes also two additional items, i.e. 'Control over pain' and 'Ability to decrease pain'. The Swedish version of CSQ has shown good reliability in evaluating pain patients' coping strategies [45].

Short Form 36 (SF 36) is used to describe patients health related quality of life [47-49]. The SF 36 is a 36-item questionnaire, and has been found to be reliable, valid and responsive [48,49]. Following the recommended procedure, the items are converted to eight scales representing generic health concepts. These eight health concepts are Physical Functioning (PF), Role function – Physical aspect (RP), Bodily Pain (BP), General Health perception (GH), Vitality (VT), Social Functioning (SF), Role function – Emotional aspect (RE), and Mental Health (MH). The score for each of the eight scales ranges from 0 to 100. A higher score indicates better health in that aspect. Short Form 36 (SF 36) is also used for calculation of QALYs and for cost-utility analyses [49,50].

Exercise Diary is used in order to measure compliance with standard exercise and check the physical activity level.

### Data management

Intention-to-treat analysis [51] with per protocol method is used in all the calculations. Per protocol method is chosen because of the nature of the acute condition, i.e. all patients are expected to get better. Descriptive statistics and appropriate multivariate analyses (Linear Mixed Models) will be used to decrease type I error in analyses of group differences over time. The Linear Mixed Models permit correlations (due to repeated measuring over time) between variables and can handle the missing values in a proper way.

#### Cost accounting analyses

Costs have to be integrated with obtained quality and quantity of life in order to measure the significance of an intervention on a person and society. Cost-utility analyses, which are a type of cost-effectiveness analyses estimate cost per QALY (Quality-adjusted life year) gained and are based on health-related quality of life measures [52,53]. Treatment costs will be evaluated for each type of management. Cost utility analysis at one and two-year follow-ups are based on SF 6D (derived from SF 36) and costs measured with cost-diary. The mean for health-related quality of life measure at baseline, 12 and 24 months are determined [50]. The mean gain is then calculated. Also cost-per-quality-adjusted-life-year (QALY) will be evaluated for clinical benefits for total lifetime costs from each group.

### Study size and Power calculations for the sample

The power calculation is based on repeated three-group analysis. Since there are no previous studies to compare with, the power calculation is based on hypothetical reasoning of effects. Upon completion of treatment, all groups will be assumed to have achieved 75 per cent improvement in the main outcome variable, Pain Disability Index, compared with the baseline levels. We assume that at the one-year follow-up, standard-treatment group will have lost 75 percent of improvement and group 1 and 2 25 percent of improvement. This gives a long-term effect size which is >.80. At a significance level of .05, a two-tailed analysis requires 60 people in each group to attain a statistical power of .90. Time needed to include approximately 200 patients (including possible drop-outs) with these inclusion criteria is expected to take at least 1,5 to 2 years according to empirical experience.

### Ethical aspects

The study has been approved by the Research Ethics Committee at Uppsala University, Uppsala, Sweden (01–229). In the IT-based intervention study it is particularly important to consider certain aspects involving confidentiality, security, authenticity, and "computer access" as an inclusion criterion. Confidentiality and security requirements must be met by means of technical solutions to ensure no unauthorized access to the treatment site. Such solutions exist and have been used in other intervention studies. Validation that it actually is the person included in the study who is responding is another ethical issue. Individuals in IT-based intervention will be enrolled via the emergency ward where all personal information is available. Comparing self-reported information with the emergency ward's data increases the validity of responses. The inclusion criterion "access to a computer" may lead to the exclusion of several potential participants, which involves limitations to the generalization of results. However, the average age of patients with WAD as a group is about 40, which means that most have access to a computer. In order to check how many patients are excluded for this reason, the number of patients fulfilling all other criteria but who lack computer access should also be reported. Study participants will have easier access to information and be more easily able to ask questions and receive answers about the study using information technology.

## Discussion

Whiplash injuries have increased considerably over the past twenty years and have become a public health problem entailing a substantial economic burden on society. Women are over-represented among the injured and among those who suffer persistent problems. Early effective care during the acute phase is crucial in the development of future symptoms. The challenging prevention of chronic whiplash associated symptoms may be possible through tailored behavioural medicine self-help strategies. Also, it is necessary to study with an experimental design if the, in many studies seen psychosocial predictors for long-term disability can be modified in patients with WAD. Further, using the Internet increases availability of the treatment method that is not dependent of therapist's office hours. Results from this project will be important in the planning of future care of WAD patients within emergency medical services and primary care.

Cost analysis is important because health care resources are limited. Decisions must be made about where time, effort, and money should be focused. Therefore, the costs of implementing different interventions must be considered along with their benefits. Short and long-term costs for patients and the health care system from the Internet-based intervention and the face-to-face group therapy programme as well as for the standard care group will be calculated in order to determine strategies for future cost-effective care of WAD patients.

Within the framework of this project, we will develop, broaden and evaluate current physical therapy treatment methods for acute WAD. Such clinically useful treatment methods will then be available for use in both the emergency and primary care settings. The project will contribute to the creation of a modern, cost-effective behavioural medicine approach to rehabilitation in WAD. The results of this study will answer an important question; on what extent and how should these patients be treated at acute stage and how much does the best management cost in QALYs.

## Abbreviations

WAD: Whiplash Associated Disorders; QALY: Quality Adjusted Life Years

## Competing interests

The authors declare that they have no competing interests.

## Authors' contributions

Authors AS, AB and PÅ; 1) have all contributed to conception and design, and acquisition of data; 2) have been involved in drafting the manuscript and revising it critically for important intellectual content; and 3) have given final approval of this version to be published.

## Pre-publication history

The pre-publication history for this paper can be accessed here:



## Supplementary Material

Additional file 1 **Description and content of the treatment modules and the phases**. A description and content of the treatment modules and the phases, intervention strategies, and examples for homework assignments of the individually tailored behavioural medicine intervention for internet-based- and face-to-face groups. Click here for file
