# Supplementary material for: A three-group study, internet-based, face-to-face based and standard- management after acute whiplash associated disorders (WAD) – choosing the most efficient and cost-effective treatment: study protocol of a randomized controlled trial
Source: BMC Musculoskelet Disord. 2009 Jul 22;10:90. doi: 10.1186/1471-2474-10-90 (PMC2722568; doi:10.1186/1471-2474-10-90)
Supplement: Additional file 1 — Description and content of the treatment modules and the phases. A description and content of the treatment modules and the phases, intervention strategies, and examples for homework assignments of the individually tailored behavioural medicine intervention for internet-based- and face-to-face groups. [file 1471-2474-10-90-S1.pdf]

Treatment Module/ Face-to-face sessions	Phases and components	Clinical strategies	Examples of homework assignments
A	**1. Problem- and goal identification**<br>Introduction: purpose, responsibilities	The Patient Goal Priority Questionnaire. Creation of a goal priority list including:<br>- Behavioral goal identification<br>- Goal ranking (importance, frequency, level of difficulty)<br>- Activity level, perceived self-efficacy, fear of performance, readiness to change, and expectations for treatment<br>Pain history<br>Physical assessment	To complete, modify and reflect over the goal priority list
B	**2. Self-monitoring**	Selection of first target for treatment<br>Tailoring of a self-monitoring diary for prospective recording (e.g. activity, situation, pain, cognitions, motor behavior, consequences)	To monitor activity in everyday-life according to agreed quota
B	**3. Functional behavioral analysis**<br>Goal setting	Introduction and discussion of hypothesized physical, cognitive, behavioral, psychosocial determinants of disability<br>Operationalization of goals and intermediate objectives	Continuing self-monitoring<br>To engage in a physical activity of own choice

**C, D, E**	**4. Basic skills acquisition**	Skills constituent to the first target are introduced, trained, and recorded	To practice basic exercises<br>To record dose, perception of success and barriers for exercising in an exercise diary
**C, D, E**	**5. Applied skills acquisition**	Basic skills are merged to shape adequate behaviors	To apply skills and practice activities in everyday-life
	contrived situations	Supervision in a contrived situation at the clinic	
	everyday-life situations	Agreement of quota for applied activities training in everyday-life	
**F**	**6. Generalization**	Subsequent goals on the priority list are targeted<br>Continuing functional behavioral analysis<br>Skills acquisition	See above<br>To extend the application of skills to new activities and situations
**G**	**7. Maintenance and relapse prevention**	Strategies for maintenance of skills are discussed<br>Future risk situations are identified, management strategies are discussed and documented	To document problem-solving strategies
